# Neuropathogenesis of Usutu virus-associated disease in Eurasian blackbirds (*Turdus merula*) involves apoptosis through the extrinsic pathway

**DOI:** 10.1007/s13365-025-01302-6

**Published:** 2026-03-02

**Authors:** Giuseppe Giglia, Gianfilippo Agliani, Bas B. Oude Munnink, Reina S. Sikkema, Maria Teresa Mandara, Christine Fast, Marleen Gosens, Eefje J. A. Schrauwen, Erwin de Bruin, Andrea Gröne, Judith M. A. van den Brand

**Affiliations:** 1 Present Address: Department of Veterinary Medicine, University of Perugia, Perugia, 06126 Italy; 2 Division of Pathology, Faculty of Veterinary Medicine, Utrecht University, Utrecht, 3584 CL the Netherlands; 3 Department of Viroscience, Erasmus University Medical Center, Rotterdam, 3000 CA the Netherlands; 4 Institute of Novel and Emerging Infectious Disease, Friedrich-Loeffler Institut, Isle of Riems, D-17493 Germany; 5 Academy for Technology of Health and Environment, Avans University of Applied Science, Breda, 4817 LL the Netherlands; 6 Dutch Wildlife Health Centre, Utrecht University, Utrecht, 3584 CL the Netherlands

**Keywords:** Usutu virus, Extrinsic apoptosis, Avian, Neuropathology, Cell-death

## Abstract

Usutu virus (USUV) is a zoonotic arbovirus causing systemic and neuroinvasive diseases in birds and flu-like or sporadic neuroinvasive disease in humans. USUV-associated encephalitis is morphologically well-characterized in birds, while the mechanisms of cell-damage in spontaneously infected birds remain poorly understood. For the closely related West Nile virus, apoptosis significantly contributes to the pathogenesis of othoflavivirus-induced neuropathology. This study investigates the involvement of apoptosis in USUV-associated encephalitis in Eurasian blackbirds (*Turdus merula*). To determine the apoptosis involvement, immunohistochemistry for cleaved-caspase-3 (CC3), TUNEL-assay, and RT-qPCR to compare mRNA expression levels of caspase 8 (extrinsic pathway) and caspase 9 (intrinsic pathway) were performed on brains of USUV-infected (*n* = 26) and USUV-uninfected (*n* = 16) blackbirds, as well as Africa 3 and Europe 3 USUV-lineages. The results showed a higher CC3 expression in USUV-infected (m = 231,96 ± 117,033) compared to uninfected blackbirds (m = 22,00 ± 13,31) (*p* < 0.001). Similarly, higher TUNEL-positive cell counts were detected in USUV-infected (m = 197,00 ± 83,56) compared to uninfected blackbirds (m = 19,00 ± 16,08) (*p* < 0.01). No significant differences were detected comparing Africa 3 and Europe 3 lineages. Additionally, both caspases were upregulated in USUV-infected blackbirds. Caspase 8 was in average 4,37 ± 0,89 (CI α = 0,05) times higher in USUV-infected blackbirds (*p* < 0.0001), while caspase 9 expression was in average 1,82 ± 0,89 (CI α = 0,05) times higher in USUV-infected blackbirds (*p* < 0.01). Although both apoptotic pathways are involved, the extrinsic pathway is notably more active in USUV-infected blackbirds, highlighting its role in USUV-associated brain damage.

**Supplementary Information:**

The online version contains supplementary material available at 10.1007/s13365-025-01302-6.

## Introduction

Usutu virus (USUV) is a zoonotic mosquito-borne arbovirus from the *Flaviviridae* family, primarily transmitted by *Culex* spp. mosquitoes. Its environmental persistence is due to a bird-mosquito-bird transmission cycle (Agliani et al. 2023b). Birds, particularly passerines such as Eurasian blackbirds (*Turdus merula*), and birds of prey, such as Great Gray Owls (*Strix nebulosa*), have been commonly reported as highly susceptible to USUV-associated disease development, leading to significant mortality (Hubálek et al. 2014; Lecollinet et al. 2016; Rijks et al. 2016; Garigliany et al. 2017; Giglia et al. 2021; Agliani et al. 2023b). Five USUV European (Europe 1 to Europe 5; EU1-EU5) and three African (Africa 1 to Africa 3; AF1-AF5) lineages have been identified (Vilibic-Cavlek et al. 2020). In Europe, both European and African lineages circulate, with some European countries having a higher circulation of one lineage over others (i.e., the Netherlands reports a higher circulation of the Africa 3 lineage) (Vilibic-Cavlek et al. 2020; Munnink et al. 2020). For some of these lineages, data on disease severity are available in literature, reporting a similar virus-associated damage (Zecchin et al. 2021; Giglia et al. 2021; Störk et al. 2021). In humans, USUV generally causes mild flu-like symptoms, though sporadic cases of neuroinvasive disease have been reported in Europe (Giglia et al. 2024). As for other orthoflaviviruses, the morphology of the central nervous system damage from USUV infection involves acidophilic neuronal necrosis, neuronophagia, glial nodules and inflammatory perivascular cuffs (Agliani et al. 2023b). For the related West Nile virus (WNV), neuropathologic lesions have been associated with molecular mechanisms of cell death (Samuel et al. 2007; Kleinschmidt et al. 2007; Kobayashi et al. 2012). Apoptosis, as a programmed mechanism of cell death, plays a key role in orthoflavivirus infection, involving both intrinsic and extrinsic pathways (Ghosh Roy et al. 2014; Okamoto et al. 2017; Pan et al. 2021). The intrinsic pathway is triggered by damage to mitochondria, which release cytochrome C and apoptosis-activating factor. These, together with pro-caspase-9, form the apoptosome. The apoptosome subsequently activates caspase-9, initiating the apoptotic cascade. The extrinsic pathway, on the other hand, is induced by external “death signals”, mediated by ligands binding to death receptors on the cell surface. This ligand-receptor binding leads to trimerization of the death receptor and activation of the DISC protein complex, which subsequently activates caspase-8. In the final steps, caspase-9 in the intrinsic pathway and caspase-8 in the extrinsic pathway cleave and activate downstream caspases, initiating a cascade that ultimately leads to caspase-3 activation and cell death (Santagostino et al. 2021). Orthoflaviviruses can adopt different mechanisms to activate these apoptotic pathways, modulating the response of the infected cell. However, when the viral load is extremely high, or if apoptotic pathways are blocked, the cell can be induced to die through necrosis or other alternative mechanisms of cell death (Ghosh Roy et al. 2014).

A caspase 3-dependent apoptosis has been described as a main WNV-associated mechanism of neuronal cell damage (Samuel et al. 2007). Experimental research additionally suggests that caspase inhibitors mitigate WNV-induced neuronal cell damage (Kleinschmidt et al. 2007).

For USUV, the role of caspase-dependent apoptosis has been demonstrated only in experimental mammalian models, while no molecular data are available for naturally infected (wild) birds (Weissenböck et al. 2004; Clé et al. 2020). This study investigated the involvement of apoptosis in USUV-associated encephalitis in naturally infected Eurasian blackbirds, examining aspects of the molecular pathways involved.

## Materials and methods

### Animals and virology

All the Eurasian blackbirds (*Turdus merula*) included in this study were collected and submitted for surveillance to the Dutch Wildlife Health Centre (DWHC) at Utrecht University in Utrecht, the Netherlands. A selection of animals was made specifically for this study from the cohort of animals examined at the DWHC and included in previous studies (Giglia et al. 2021; Agliani et al. 2023a). Inclusion criteria for the cases included in this study were: the species (being a Eurasian blackbird), the availability of formalin-fixed paraffin embedded and frozen brain tissue (-80 °C) of good quality for a carcass code 2 (low autolytic status of the carcass at examination) (McAloose et al., 2018) and, for USUV-uninfected birds, the complete absence of any lesion in the central nervous system. The animals were divided in two groups based on the virological status (infected and uninfected based on USUV RT-qPCR results of brain tissue). Virological status and virus lineages were retrieved from the repository of internal reports and determined as previously reported (Jöst et al. 2011; Oude Munnink et al. 2019; Giglia et al. 2021). The mean cycle threshold (Ct) value in infected blackbirds was 20.13 ± 3.66. Based on the criteria mentioned above, a total of forty-two (*n* = 42) Eurasian blackbirds were selected from previous studies and included in this study, including twenty-six (*n* = 26) USUV-infected blackbirds (20 with the Africa 3 lineage and 6 with Europe 3 lineage) and sixteen USUV-uninfected blackbirds (*n* = 16). The study included 14 males, 17 females, and 11 individuals of undetermined sex. Age classification identified 25 mature individuals and 16 juveniles, while age data were unavailable for one case.

### USUV and cleaved-caspase-3 immunohistochemistry and terminal deoxynucleotidyl transferase dUTP Nick End Labeling (TUNEL)-assay

Brain tissue represented by random samples of the avian hemispheres (telencephalon) collected during necropsies were fixed in 10% neutral buffered formalin. To test the hypothesis that Usutu virus (USUV) infection involves apoptosis as mechanism of cell death in the pathogenesis of the disease, the expression of cleaved-caspase-3 (CC3) in Immunohistochemistry (IHC) in USUV-infected blackbirds was compared with USUV-uninfected blackbirds. Additionally, anti-Usutu virus IHC was performed to identify the distribution, localization and amount of virus antigen in the tissue, in association with the damage. Briefly, after routine processing and paraffin embedding, 3 μm consecutive sections were cut. Sections were deparaffinized in xylene and rehydrated in decreasing concentrations of alcohol. Antigen retrieval was performed in citrate buffer (pH6). Endogenous peroxidases were blocked with 3% H_2_0_2_. Sections for the CC3 investigation on IHC were incubated with a polyclonal antibody (1:100 dilution; Abcam, Ab2302), while for Usutu virus, sections were incubated with an anti-USUV serum (polyclonal antibody U433, Friedrich-Loeffler Institut, Isle of Riems, Germany) (1 h; 1:1500), as previously reported (Giglia et al. 2025). Subsequently, slides were incubated with biotinylated goat anti-rabbit IgGs. Sections were then incubated with streptavidin horseradish-peroxidase. Positivity (immunoreactivity) was visualized with AEC chromogen (Abcam) counterstained with Haematoxylin. In addition, a TUNEL (Terminal deoxynucleotidyl transferase dUTP Nick End Labeling) assay, was conducted on a subset of cases based on the availability of tissue (*n* = 6 USUV-infected blackbirds, *n* = 4 USUV-uninfected blackbirds) at the Academy for Technology of Health and Environment, Avans University of Applied Science (Breda, the Netherlands). The TUNEL assay was performed using a kit (Abcam, ab206386). The staining method was performed accordingly to manufacturer’s guidelines. Due to variability in the avian brain sample size, CC3, USUV and TUNEL-positive cells were counted, blindly from the infection status, in a standard area of 2.37mm^2^ (FN 22; 400x) using an optic microscope (Olympos BX53), thereby minimizing potential bias associated with whole-slide cell counts and enabling consistent comparison across specimens. A mean (m) value and standard deviation (sd) for each parameter was defined for both the groups. Although USUV antigen detection on immunohistochemistry and its scoring with ordinal grades has been previously reported for these cases (Giglia et al. 2021), the present study employed the pure quantitative cell-counts, to directly correlate viral antigen–positive cells with CC3–positive cells. Regarding the USUV antigen and CC3 spatial localization, this was performed assessing the areas overlap in consecutive tissue sections. A co-localization approach was not feasible due to antibody species constraints as both, the anti-USUV and the anti-CC3 primary antibodies are derived from rabbits.

### Evaluation of different pathways by caspases 8 and 9 RT-PCRs

RT-PCRs were performed to evaluate the mRNA expression of the caspase 8 and caspase 9 genes involved in the two main apoptotic pathways. Total RNA was extracted from − 80 °C frozen brain tissue samples using a commercial kit and following the manufacturer’s protocol (RNeasy Plus mini kit, Qiagen), remaining DNA was removed using Turbo DNA free kit (Invitrogen). Primers for GAPDH (the reference gene used), caspase 8 and caspase 9 avian genes were designed on conserved regions of those genes and a RT-PCR was performed (details in the Supplementary material). Normalized gene expression was calculated according to the 2^−∆∆ct^ method from the Ct value, where the Ct of GAPDH was used as reference gene and caspase 8 and caspase 9 as target genes.

### Statistical analysis

All statistical analyses were performed using R software (version 4.2.3) and statistical significance was set at *p* ≤ 0.05. Graphics were used to test assumptions. To compare the means of CC3-positive cells and TUNEL positive cells between USUV-infected and uninfected blackbirds, and between identified virus lineages, a student’s t-test was performed. To evaluate potential association of the number of USUV-positive cells and CC3-positive cells, a Spearman correlation analysis was performed. Statistical analysis was performed to compare the expression levels of caspase 8 and caspase 9 between USUV-infected and USUV-uninfected blackbirds, and between identified virus lineages with the 2^−∆∆ct^ method. To compare the difference between the two groups, a student’s t-test was performed. To evaluate the presence of a correlation between USUV RT-qPCR ct values and caspase 8 and caspase 9 RT-qPCR ct values, a Pearson’s Correlation Coefficient Test was performed.

## Results

On all the cases, IHC for USUV and CC3 was performed on brain tissue. To associate the virus presence in areas of apoptosis activation, a spatial distribution of USUV antigen and CC3–positive cells were assessed in sequential brain sections from USUV-infected blackbirds. The analysis demonstrated a partially overlapping distribution of the two antigens. USUV antigen was detected in multifocal areas of the neuroparenchyma, with variable numbers of infected cells (m = 97.04 ± 173.37) within the individual foci and as rarely scattered cells. No USUV antigen-positive cells were observed in any uninfected blackbirds. In corresponding foci, CC3-positive cells were observed (Supplemental Fig. S1). However, a higher number of CC3-positive cells was detected, these were also disseminated throughout the surrounding neuroparenchyma, outside of the foci showing USUV antigen. At higher magnification, USUV antigen was primarily localized in cells with morphological features consistent with neurons, glial and endothelial cells. CC3 immunoreactivity was observed in cells with comparable morphology. The Spearman correlation analysis showed a significant correlation between the USUV-positive cell count and the CC3-positive cell count (ρ = 0.61, *p* < 0.001).

**Fig. 1:**
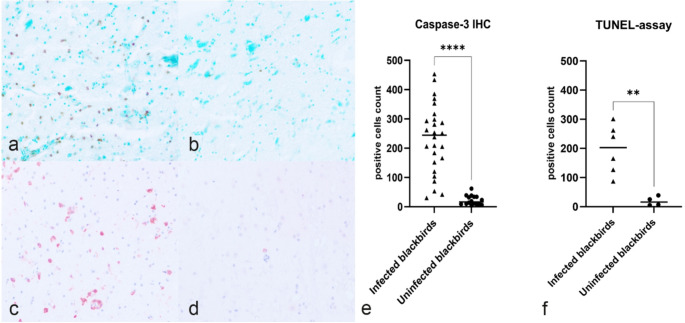
TUNEL-assay and Cleaved-Caspase-3 (CC3) immunohistochemistry (IHC) cell counts in USUV-infected and uninfected blackbirds; (**a**) Infected blackbirds show a high number of TUNEL-assay positive nuclei in cells morphologically compatible with neurons, glial and endothelial cells (TUNEL-assay; 400x) compared to (**b**) uninfected blackbirds showing rare positive nuclei; **(c)** Infected blackbirds show a significative higher number of CC3-positive cells morphologically compatible with neurons, glial and endothelial cells compared to (**d**) uninfected blackbirds; (**e**) the graph shows a significantly higher CC3-positive cell count in infected blackbirds (*p* < 0.0001; ****); (**f**) the graph shows a significantly higher TUNEL-assay positive cell count in infected blackbirds (*p* < 0.01; **)

CC3-positive cells (on IHC) and TUNEL-positive cells in USUV-infected and USUV-uninfected blackbirds are shown in Fig. 1 (Fig. 1a-d). Based on the cell-count, USUV-uninfected blackbirds showed less CC3-positive cells (m = 22,00 ± 13,31), in comparison to USUV-infected blackbirds (m = 231,96 ± 117,033). The independent sample t-test reported a statistically significantly difference in means (t(26,557) = -9,007, *p* < 0.001) (Fig. 1e). For the TUNEL-assay, USUV-uninfected birds (*n* = 4) showed less TUNEL-positive cells (m = 19,00 ± 16,08), in comparison with USUV-infected birds (*n* = 6) which showed a higher number of TUNEL-positive cells (m = 197,00 ± 83,56) with morphological features as described for USUV and CC3-positive cells. The independent sample t-test reported a statistically significant difference in means (t(5,543) = -5,079, *p* < 0.01) (Fig. 1f). No statistically significantly difference was observed between the lineages (*p* > 0.05).

**Fig. 2:**
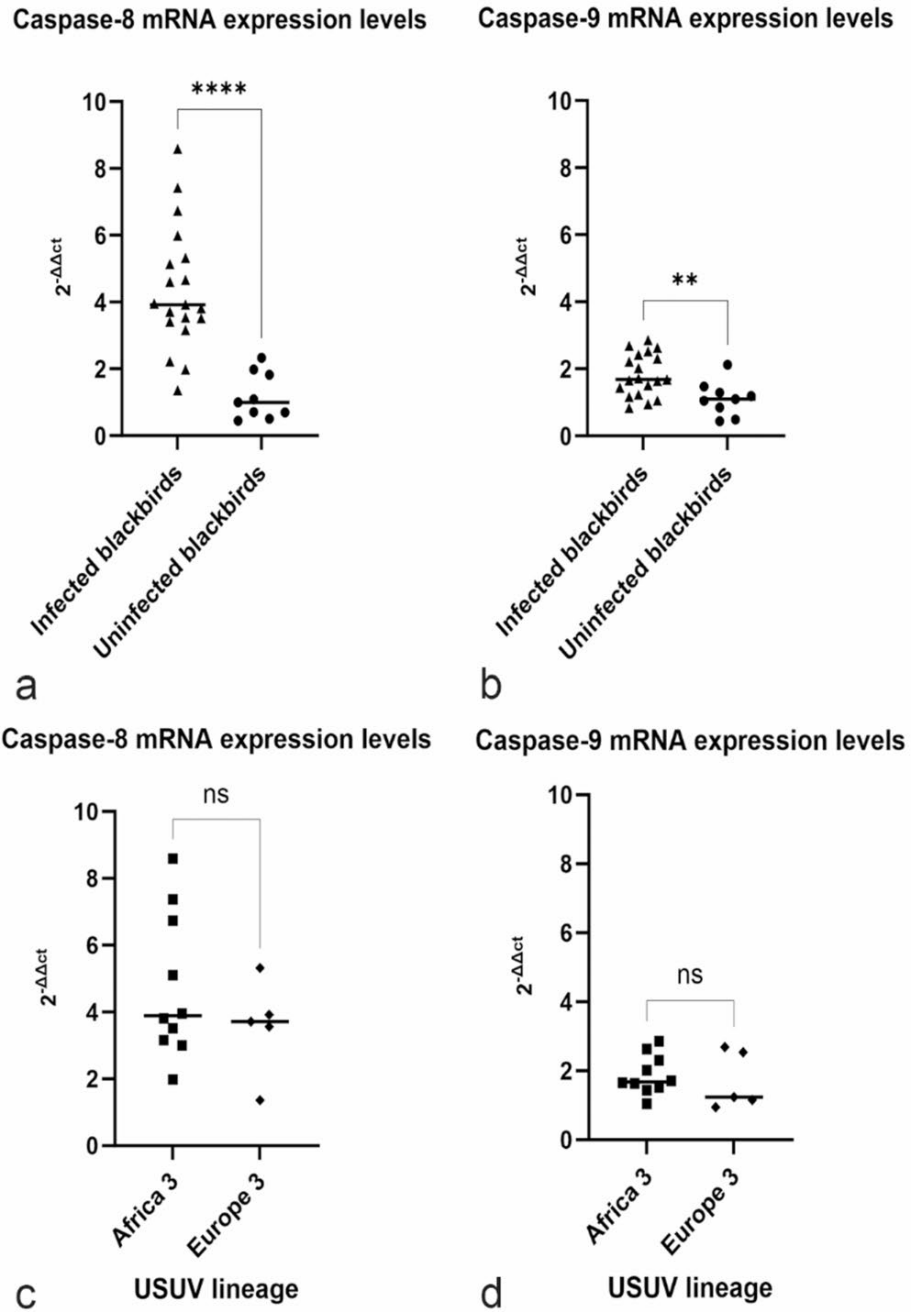
Relative caspase 8 and caspase 9 gene expression of Usutu-infected compared to uninfected blackbirds and in two identified lineages calculated with the 2^−∆∆ct^ method with GAPDH as housekeeping gene used for normalization; (**a**) Caspase 8 expression levels in USUV-infected blackbirds with an average of 4,37 ± 0,89 (CI α = 0,05) times higher compared to the uninfected blackbirds (*p* < 0.0001; ****); (**b**) Caspase 9 expression levels with an average of 1,82 ± 0,89 (CI α = 0,05) times higher compared to the uninfected blackbirds (*p* < 0.01; **); (**c**) Caspase 8 expression levels in USUV-infected blackbirds with no significant differences in Africa 3 infected blackbirds compared to Europe 3 infected blackbirds (*p* > 0.05); (**d**) Caspase 9 expression levels in USUV-infected blackbirds with no significant differences in Africa 3 infected blackbirds compared to Europe 3 infected blackbirds (*p* > 0.05)

To determine the difference in apoptosis initiation via the extrinsic and intrinsic pathways in brain tissue of blackbirds, a RT-qPCR approach was employed. Using the 2-∆∆Ct method, the differential expression of caspase 8 mRNA relative to the housekeeping gene GAPDH was calculated. The average relative gene expression of caspase 8 in USUV-infected blackbirds (*n* = 19) is on average 4,37 ± 0,89 (CI α = 0,05) times higher than the USUV uninfected blackbirds (*n* = 9). The results indicate a significant upregulation of caspase 8 mRNA in the brains of USUV-infected blackbirds (*p* < 0.0001) (Fig. 2a). Additionally, upregulation of caspase 9 mRNA in the USUV-infected blackbirds compared to the USUV uninfected blackbirds was significant (*p* < 0.01). (Fig. 2b) No statistically significantly difference was observed for caspase 8 and caspase 9 mRNA in the brain between Africa 3 and Europe 3 USUV lineages (*p* > 0.05) (Fig. 2c-d). A significant correlation was observed at Pearson’s test between USUV Ct values and caspase 8 and caspase 9 RT-qPCR Ct values. The results showed a moderate positive correlation between USUV Ct and casp8 Ct (*r* = 0.594, *p* < 0.01; 95% CI [0.191, 0.826]). Similarly, a moderate positive correlation was observed between USUV Ct and casp9 Ct (*r* = 0.635, *p* < 0.01; 95% CI [0.255, 0.846]).

## Discussion

This study demonstrates the involvement of apoptosis in USUV-associated encephalitis in naturally infected Eurasian blackbirds by examining certain aspects of the molecular pathways involved. Forty-two Eurasian blackbirds (*Turdus merula*) were selected from previous morphological studies on USUV-related disease, of which 26 were infected with USUV and 16 were uninfected. Immunohistochemistry for CC3 showed a significant difference in the number of positive cells between USUV-infected and uninfected blackbirds: the USUV-infected blackbirds showed significantly more cells positive for CC3 than the uninfected blackbirds. The TUNEL assay, confirmed significantly more positive cells in infected blackbirds than in uninfected ones. Whereas no knowledge is reported on the mechanisms of apoptosis in naturally infected animals, apoptosis has been described for USUV only on experimental in-vitro and in-vivo studies (Weissenböck et al. 2004; Clé et al. 2020). Similarly, the involvement of apoptosis has been described for other orthoflaviviruses with variable pathogenicity compared to USUV (Riccetti et al. 2020). WNV for instance has a well-established association with apoptosis induction and demonstrates a greater cytopathic effect in human neural stem cells compared to USUV (Riccetti et al. 2020). Future studies comparing differences in apoptosis induction between USUV and WNV in birds are essential and could provide insights into differences in the severity of the neurological damage. Additionally, since USUV and WNV share similarities, by comparing these viruses, researchers could explore potential interventions and refine strategies for antiviral therapies, ultimately improving our ability to manage and treat flaviviral diseases.

Positive cells for CC3 and TUNEL-assay detected in the current study were morphologically compatible with neurons, glial cells, and endothelial cells. All these cell types have been reported in Eurasian blackbirds as susceptible to USUV infection by detection of cytoplasmic virus antigen by IHC (Agliani et al. 2023b).

For this reason, a potential direct involvement of USUV in the signaling of apoptosis in those cells should be considered and further evaluated. For WNV for instance, the role of some miRNAs (miR-451a and Hs_154rs) has been linked to orthoflavivirus-induced neuronal apoptosis. Similar data are not available for USUV-related damage (Smith et al. 2012; Chakraborty and Basu 2022). Potential limitations of the study related to the lack of currently validated cell-type–specific antibodies in Eurasian blackbirds’ tissues, which imposed the reliance on standard histomorphological criteria for the cellular identification. Further studies with cell-type–specific antibodies validated in blackbirds are needed to further confirm the cellular types involved in the USUV infection and related damage (Webster et al. 2021).

Regarding the localization of USUV-antigen and cleaved caspase-3, although single-cell co-localization was not feasible due to antibody species constraints, their partially overlapping distribution in sequential tissue sections supports an association between viral presence and apoptosis activation. Furthermore, although significantly correlated, the higher number of CC3–positive cells compared with USUV-positive cells might suggest the contribution of indirect mechanisms affecting the surrounding tissue and cell death extending beyond directly infected cells. In this context, the virus-induced glial cells damage disrupting the tissue homeostasis, as well as the detrimental neuroinflammation or the endothelial cell damage in neuroparenchymal vessels, may play a role triggering secondary cascades of tissue and cellular injury thereby amplifying tissue damage largely beyond direct the virus–cell interactions (Klein et al. 2019). Cleaved-caspase-3 IHC and TUNEL-assay are valuable tools for detecting apoptosis, but they only provide a partial picture of the cell death process. While these methods confirm the presence of apoptotic cells and indicate that cell death is occurring, they do not reveal the upstream mechanisms, and which apoptotic molecular pathway is being triggered by the virus. Regarding the molecular pathways, the current study shows in USUV naturally infected blackbirds the upregulation of caspase 8 and caspase 9 in infected blackbirds compared to uninfected birds, assessed by RT-qPCR. This finding suggests the activation of both, the extrinsic and intrinsic pathways of apoptosis in USUV-infected blackbirds. The upregulation of caspase 8 was stronger in infected blackbirds compared to caspase 9. This suggests that, similarly to WNV, USUV infection could simultaneously activate both apoptotic pathways, contributing to the observed brain damage, and that the extrinsic (caspase 8-mediated) pathway might have a potential prevalent role (Kleinschmidt et al. 2007). For WNV, both pathways have been recorded, and many viral factors have been associated with the initiation of one of the two pathways. For instance, the viral protein NS3 promote the transcription of TNF-α, which is a pro-apoptotic signal for the initiation of the extrinsic pathway (Ramanathan et al. 2006; Okamoto et al. 2017; Pan et al. 2021). By understanding these interactions, antiviral therapies that target specific viral proteins to prevent excessive cell death or uncontrolled viral spread could be investigated.

To compare the involvement of apoptosis in cases of USUV-infection with different lineages, six samples infected with the USUV Europe 3 lineage and 20 samples with the USUV Africa 3 lineage were included, reflecting the relative scarcity of Europe 3 infections in our dataset, as this lineage is less frequently detected in the Netherlands compared to Africa 3 (Munnink et al. 2020). Although in some Africa 3-infected cases a higher CC3 cell count and caspase 8 mRNA expression was seen, no significant differences were observed between the Europe 3 and the Africa 3 lineages, suggesting a similar pathogenicity and molecular pathways involvement, as seen on the avian literature knowledge on the severity of virus-associated lesion in naturally and experimentally infected blackbirds (Giglia et al. 2021; Agliani et al. 2025). This result is in contrast with the data derived from infected mice and neuronal cell cultures, where a lineage-dependent differences in the virulence profiles (Clé et al. 2021).

Regarding the role of apoptosis in orthoflavivirus infections, both a beneficial and a harmful effect have been reported (Pan et al. 2021). On one side, apoptosis allows a rapid infection of surrounding cells and thus increases the rate of infection in the tissue without eliciting a strong inflammatory response. Specifically, an “apoptotic mimicry” mechanism has been proposed (Amara and Mercer 2015). After the cell has undergone apoptosis, the apoptotic bodies containing the virus can be phagocytosed by phagocytic cells and may remain infectious for other cells without entering via the extracellular space and avoiding contact with immune cells for clearance. On the other side, the apoptosis is still considered one of the mechanisms that the cell and the immune system use to counteract the viral infection (Pan et al. 2021). The detection of the apoptosis involvement in USUV-associated encephalitis in naturally infected blackbirds lead the way to further investigations aiming to understand how the virus action is molecularly link to this cell-death mechanism and propose new therapeutic targets that could limit CNS damage during USUV-associated disease.

## Conclusions

This study demonstrates the involvement of apoptosis in the USUV-associated encephalitis in naturally infected Eurasian blackbirds. The significative higher number of positive cells for cleaved caspase-3 immunohistochemistry and TUNEL-assay, indicate engagement of a caspase-dependent cell death pathway in this viral disease. The partial overlap of USUV-antigen and CC3 in tissue foci, as well as the disseminate CC3-positive cell presence suggest both, a direct and an indirect involvement of USUV in the caspase-dependent cell death. A higher expression of caspase 8 is suggestive for the extrinsic pathway involvement in USUV-associated encephalitis. No differences between the Europe 3 and the Africa 3 lineage were observed in the examined blackbirds. Further studies should evaluate the direct and indirect effects of the Usutu virus and related disease in determining the apoptotic cell death in USUV-associated encephalitis and disease in other organs. Additionally, studies focusing on the identification of the affected cells by using specific cellular markers could elucidate the pathogenesis of apoptosis in neurological disease related to USUV infection.

## Supplementary Information

Below is the link to the electronic supplementary material.

Supplementary Material 1

## Data Availability

No datasets were generated or analysed during the current study.
