# Supplementary material for: Neuropathogenesis of Usutu virus-associated disease in Eurasian blackbirds (Turdus merula) involves apoptosis through the extrinsic pathway
Source: J Neurovirol. 2026 Mar 2;32(2):9. doi: 10.1007/s13365-025-01302-6 (PMC12953318; doi:10.1007/s13365-025-01302-6)
Supplement: Supplementary file 1 — Supplementary Material 1 [file 13365_2025_1302_MOESM1_ESM.docx]

**Supplemental Material**

For the detection of mRNA quantities of target genes GAPDH, Caspase 8 and Caspase 9 from blackbirds, primers were designed using Bioedit (T. Hall, 1999, version 7.0.9.0). As most sequence information regarding the genome of the blackbird is unknown, we searched for known bird sequences of the target genes (see table S1). Based on the alignment per gene, primers were designed on conserved regions showing minimal primer dimers. Primers were tested using dilution series of extracted RNA from blackbirds to a range of annealing temperatures and for specificity by melting curve analysis using SYBR green and 1.5 percent agarose gel electrophoresis. Optimal primers for each gene are shown in table S2. For Caspase 8 it was not possible to find a primer combination without any side product, therefor it was decided to use a probe detection in combination with a primer pair having minimal side products.

RT-PCR was done using iTaq SYBR Green One-Step Kit (Biorad; GAPDH and Caspase 9) or the TaqMan™ Fast Virus 1-Step Master Mix (Applied Biosystems; Caspase 8) using a CFX connect real time PCR machine (Bio-rad). For GAPDH the cycling protocol started with an incubation at 50 ⁰C for 10 minutes followed by 95 ⁰C for 1 minute. After the reverse transcriptase step, 45 cycles of amplification at 95 ⁰C for 10 seconds and 60 ⁰C for 30 seconds followed, after which a melting curve analysis was done from 65 to 95 ⁰C (increment of 0.5⁰C for 2-5 seconds per step). Detection of Caspase 9 was done the same except an additional annealing step of 55 ⁰C for 15 seconds which was included between the denaturation (95 ⁰C) and elongation step (60 ⁰C) during each cycle. Reverse transcription for Caspase 8 started with 50 ⁰C for 5 minutes followed by 95 ⁰C for 20 seconds. This was followed by 45 amplification cycles at 95 ⁰C for 15 seconds, 55 ⁰C for 30 seconds and 60 ⁰C for 30 seconds.

Table S1; Sequences used for the design of the primers targeting GAPDH, Caspase 8 and Caspase 9 gene.

| Species | Name | GAPDH | Casp8 | Casp9 |
| --- | --- | --- | --- | --- |
| *Catharus ustulatus* | Swainson's thrush | XM_033053117.1 PREDICTED | XM 033065450.1 PREDICTED | XM_042779967.1 |
| *Parus major* | Great tit | XM_033517093.1 | XM 033515951.1 PREDICTED | XM_033519336.1 PREDICTED |
| *Sturnus vulgaris* | Common Starling | XM_014884173.1 | XM 014879454.1 PREDICTED | XM_014881727.1 PREDICTED |
| *Onychostruthus taczanowskii* | White-rumped snowfinch |  | XM 041403786.1 PREDICTED | XM_041419572 PREDICTED |
| *Pyrgilauda ruficollis* | Rufous-necked snowfinch |  | XM 041472936.1 PREDICTED | XM_041463468.1 |
| *Corvus brachyrhynchos* | American crow | XM_017731658.1 PREDICTED | XM 008629734.2 PREDICTED | XM_017735079.1 PREDICTED |
| *Gallus gallus* | Chicken | NM_204305.2 | NM 204592.4 | XM_424580.7 PREDICTED |
| *Ficedula albicollis* | Collared flycatcher |  | XM 005049178.2 PREDICTED | XM_005057750.2 |
| *Taeniopygia guttata* | Sunda zebra finch |  | XM 012574963.4 PREDICTED | XM_041720341.1 PREDICTED |
| *Catharus fuscescens* | Veery |  | VWYD01000180.1 |  |
| *Cygnus atratus* | Black Swan | XM_035571540.1 PREDICTED | XM 035537076.1 PREDICTED | XM_035557436.1 PREDICTED |
| *Cygnus olor* | Mute Swan | XM_040573143.1 PREDICTED | XM 040562455.1 PREDICTED | XM_040533286.1 PREDICTED |
| *Gavia stellata* | Red-throated loon | XM_009819508.1 PREDICTED | XM 009809594.1 PREDICTED | XM_009820903.1 PREDICTED |
| *Haliaeetus albicilla* | White-tailed eagle | XM_009923216.1 PREDICTED |  |  |

Table S2; primers used for mRNA detection in blackbirds

| Target gene | Sequence (5`-3`) | | Size (bp) |
| --- | --- | --- | --- |
| Caspase 9 | Forward | ACGAGCCATATTCAGTTTCC | 130 |
|  | Reverse | TGAAGAACAGTTTGGGTTTTCC |  |
| Caspase 8 | Forward | ACAGGCAACTGCTGTATGGAA | 103 |
|  | Reverse | TCCTGGAGCTTGTTCTTTGGT |  |
|  | Probe | FAM-TGGAGTAGGAACTTCACACTTC-BHQ1 |  |
| GAPDH | Forward | GGCTTTCCGTGTGCCAAC | 74 |
|  | Reverse | TCAGCAGCAGCCTTCACTAC |  |

**Supplementary References**

Hall, T.A. (1999) BioEdit: A User-Friendly Biological Sequence Alignment Editor and Analysis Program for Windows 95/98/NT. Nucleic Acids Symposium Series, 41, 95-98.

**Supplemental Figure S1**


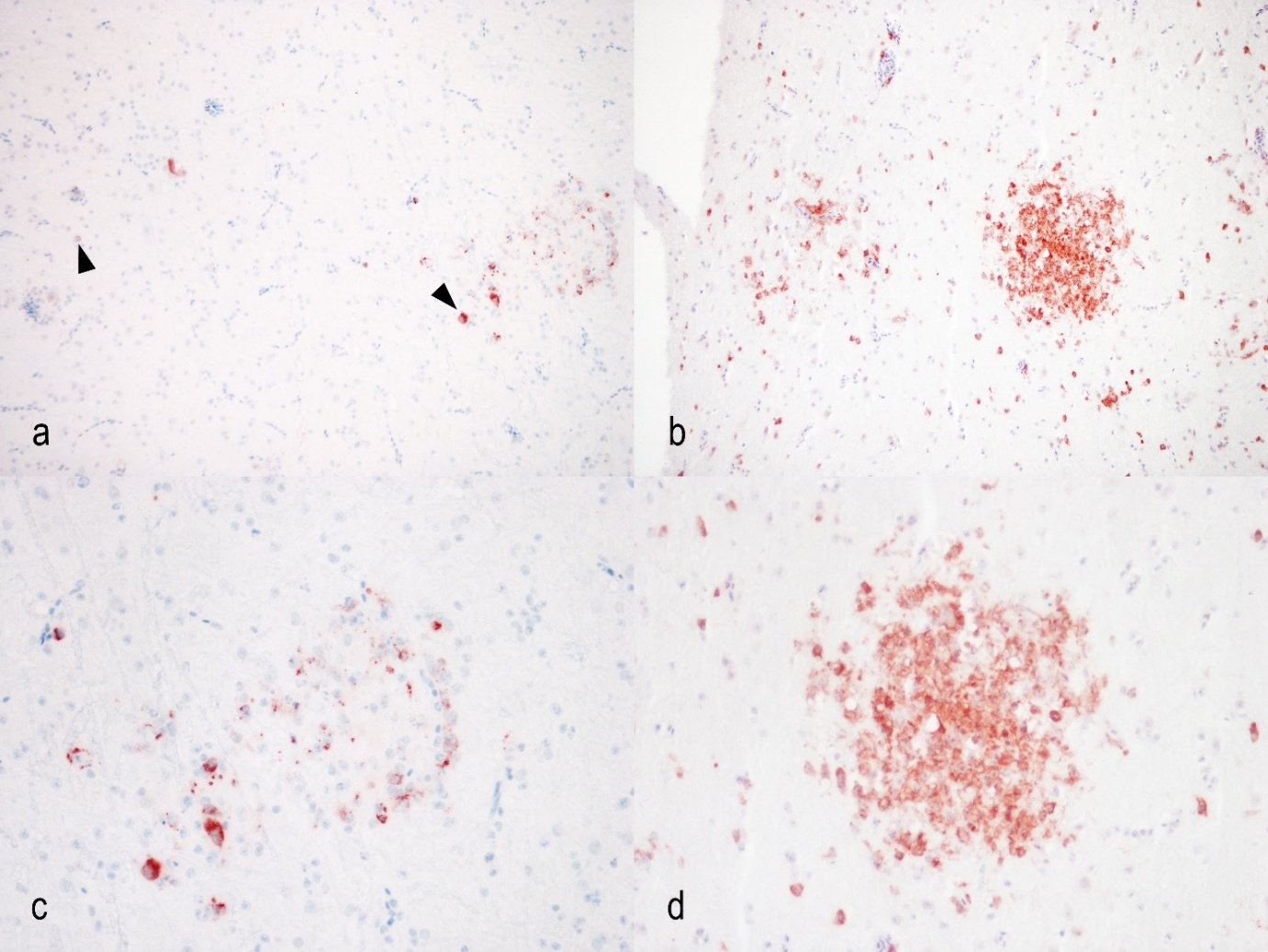


**Figure S1. Spatial distribution of Usutu virus and Cleaved Caspase-3 (CC3) antigen in immunohistochemistry (IHC) of Usutu virus (USUV) infected blackbirds**; **a)** Infected blackbirds show multifocal foci with a variable number of USUV-infected cells (anti-USUV IHC; 200x); **b)** Infected blackbirds show multifocal foci with numerous cleaved caspase-3 positive cells (anti-CC3 IHC; 200x), these are not only seen in the areas with virus antigen but also disseminated in a higher number in the neuroparenchyma; **c)** In infected blackbirds, USUV-antigen is seen primarily in neurons and glial cells (anti-USUV IHC; 400x) **d)** In infected blackbirds, cleaved caspase-3 positive cells is seen in cells with similar morphology of the ones showing USUV-antigen (anti-CC3 IHC; 400x).
